#  Controlled precision QUBO-based algorithm to compute eigenvectors of symmetric matrices

**DOI:** 10.1371/journal.pone.0267954

**Published:** 2022-05-09

**Authors:** Benjamin Krakoff, Susan M. Mniszewski, Christian F. A. Negre

**Affiliations:** 1 Department of Mathematics, University of Michigan, Ann Arbor, Michigan, United States of America; 2 Computer, Computational, and Statistical Sciences Division, Los Alamos National Laboratory, Los Alamos, New Mexico, United States of America; 3 Theoretical Division, Los Alamos National Laboratory, Los Alamos, New Mexico, United States of America; University of Southern California, UNITED STATES

## Abstract

We describe an algorithm to compute the extremal eigenvalues and corresponding eigenvectors of a symmetric matrix which is based on solving a sequence of Quadratic Binary Optimization problems. This algorithm is robust across many different classes of symmetric matrices; It can compute the eigenvector/eigenvalue pair to essentially any arbitrary precision, and with minor modifications, can also solve the generalized eigenvalue problem. Performance is analyzed on small random matrices and selected larger matrices from practical applications.

## 1 Introduction

The problem of computing eigenvectors and eigenvalues to a desired precision has many applications in science and mathematics, including web page ranking [1], planar embeddings [2], and principal component analysis [3], among many others. The recent development of new computing paradigms has led to the development of *annealing devices*, which are specialized hardware designed to solve Quadratic Binary Optimization Problems (QUBOs). Such annealers include D-Wave’s quantum annealers and Fujitsu’s Digital Annealer. This has lead to a corresponding interest in reformulating computational tasks as QUBOs and solving them using these annealing devices. This strategy has been applied to several problems including graph partitioning [4], solving polynomial equations [5], and vertex coloring [6]. Here we compute eigenvectors of symmetric matrices by solving a sequence of QUBOs, which allow the eigenvectors and eigenvalues to be found to any desired precision. A mathematically similar approach to this problem is considered in [7, 8], but accuracy is increased by increasing the size of the associated QUBO. In contrast, the proposed algorithm can compute eigenvectors to essentially arbitrary precision without increasing the size of the QUBOs, which can have as few as twice as many variables as the original eigenvalue problem. The trade-off for using small QUBOs is that more iterations are required. A similar approach is considered in Appendix C of [9], although here the effects of different parameters are more thoroughly studied, and the presentation gives a very general optimization framework. The performance data is collected using D-Wave’s Ocean Simulated Annealing (SA) package.

As mentioned, the state-of-art of QUBO eigensolver is proposed in [8]. In the aforementioned work, the only way of increasing precision is to increase the QUBO size by increasing the number of bits required to represent the real numbers. In the hereby proposed method we can compute the eigenpair to arbitrary any precision without increasing the size of the QUBO.

The paper is organized as follows. The relevant mathematical background for symmetric matrices and use of QUBO solvers as a descent method is explained in Section 2.1. The algorithm for computing the eigenvector/eigenvalue pair is given in Section 2.3. Experimental results with various parameters and matrices are presented in Section 3, followed by the conclusion in Section 4.

## 2 Methods

### 2.1 Mathematical background

Let *A* be a symmetric matrix. A well-known consequence of the spectral theorem is that the smallest eigenvalue λ and corresponding eigenvector **v** are global minima for the Rayleigh quotient **x**^*t*^*A***x**/**x**^*t*^**x**

$$\begin{array}{c} \lambda =\underset{\|x\|=1}{\text{min}}\;x^{t}Ax,\;\;v=\underset{\|x\|=1}{\text{argmin}\;}\;x^{t}Ax \end{array}$$ (1)

The proposed algorithm uses a QUBO formulation of the problem to both obtain a good initial guess for the global minimum, and to implement an iterative descent from the initial guess. Similar to classical descent methods such as Newton Conjugate-Gradient and the BFGS algorithms [10], this algorithm requires computing, but not inverting, a Hessian matrix at each descent step. We begin with an overview of QUBOs and how they can be used to approximately solve certain constrained quadratic optimization problems.

Let {0, 1}^*m*^ denote the set of binary vectors of length *m*, and let *Q* be a symmetric *m* × *m* matrix. The combinatorial optimization problem

$$\begin{array}{c} \underset{x_{b}\in {\left\{0,1\right\}}^{m}}{\text{argmin}\;}\;x_{b}^{t}Qx_{b} \end{array}$$

is called a *quantum unconstrained binary optimization* problem, or QUBO, and it is known to be NP-hard [11]. As mentioned before, interest in casting various problems as QUBOs has increased due to the development of annealing devices, which are a class of hardware that use ideas from statistical mechanics to produce approximate solutions to a QUBO. See for example [12] or [13].

To solve a real-variable optimization problem using a QUBO, we require a method of approximating each real variable by *b* binary variables. This number *b* will be a parameter referred to as the number of bits.

Let’s start with a few concrete examples of the arithmetic involved, beginning with a demonstration of how to multiply two real numbers such as *x* = −.5 and *y* = .5 using 2 bits. Form the *precision vector* **p** = (−1, .5) with corresponding *precision matrix* $P=p^{t}p=(\begin{array}{cc} 1 & -.5\\ -.5 & .25 \end{array})$. Set $x_{b}=(\begin{array}{c} 1\\ 1 \end{array})$ and $y_{b}=(\begin{array}{c} 0\\ 1 \end{array})$ so that

$$\begin{array}{cc} & x=p\cdot x_{b},\;\;y=p\cdot y_{b} \end{array}$$ (2)

$$\begin{array}{cc} & -.25=x\cdot y=x_{b}^{t}p^{t}py_{b}=\left(1,1\right)(\begin{array}{cc} 1 & -.5\\ -.5 & .25 \end{array})(\begin{array}{c} 0\\ 1 \end{array}) \end{array}$$ (3)

Now let *Q* be a symmetric matrix, and we shall demonstrate how to compute the quadratic form $\left(x,y,z\right)Q(\begin{array}{c} x\\ y\\ z \end{array})$ using binary variables. As above, set *z* = **p** ⋅ **z**_*b*_ where **z**_*b*_ is a binary vector and *z* is the corresponding real number. We can rewrite the quadratic form as

$$\begin{array}{c} \left(x_{b}^{t},y_{b}^{t},z_{b}^{t}\right)(\begin{array}{ccc} p^{t} & & \\ & p^{t} & \\ & & p^{t} \end{array})Q(\begin{array}{ccc} p & & \\ & p & \\ & & p \end{array})(\begin{array}{c} x_{b}\\ y_{b}\\ z_{b} \end{array}) \end{array}$$ (4)

The middle three terms can be written more succinctly as (*I*_3_ ⊗ *p*^*t*^)*Q*(*I*_3_ ⊗ *p*) = *Q* ⊗ *P* where *I*_3_ is the 3 × 3 identity matrix and ⊗ is the tensor product.

Now we describe the construction in full generality. Given a *precision vector* $p=(-1,\frac{1}{2},\frac{1}{2^{2}},\frac{1}{2^{3}},\ldots \frac{1}{2^{b-1}})$ of length *b*, the set of integer multiples of $\frac{1}{2^{b-1}}$ in the interval $\left[-1,1-\frac{1}{2^{b}}\right]$ is exactly the set

$$\begin{array}{c} C_{1,b}≔\left\{p\cdot x_{b}|x_{b}\in {\left\{0,1\right\}}^{b}\right\} \end{array}$$ (5)

We use the sub-scripted **x**_*b*_ as a convention to emphasize that **x**_*b*_ is a binary vector, i.e. the subscript does not refer to the number of bits. More generally, the *n*-fold product of *C*_1,*b*_ is the set

$$\begin{array}{c} C_{n,b}≔\left\{\left(I_{n}⊗p\right)x_{b}|x_{b}\in {\left\{0,1\right\}}^{nb}\right\} \end{array}$$ (6)

where *I*_*n*_ is the identity matrix. The set *C*_*n*,*b*_ will be referred to as a discretized cube. Let *Q* be a symmetric *n* × *n* matrix, **r** an *n*-vector and suppose we want to solve the constrained quadratic programming problem

$$\begin{array}{c} \underset{x\in {\left[-1,1\right]}^{n}}{\text{argmin}\;}\;r^{t}x+x^{t}Qx \end{array}$$ (7)

To get an approximate solution to (7) using a QUBO, first replace the unit cube by a discretized unit cube to get the optimization problem.

$$\begin{array}{c} \underset{x\in C_{n,b}}{\text{argmin}\;}\;r^{t}x+x^{t}Qx \end{array}$$ (8)

Setting *m* = *nb*, **x** = (*I*_*n*_ ⊗ **p**)**x**_*b*_, and *P* = **p**^*t*^**p**, this is equivalent to the following QUBO problem:

$$\begin{array}{cc} \underset{x_{b}\in {\left\{0,1\right\}}^{m}}{\text{argmin}\;}\;r^{t}\left(I_{n}⊗p\right)x_{b} & +x_{b}^{t}\left(Q⊗P\right)x_{b} \end{array}$$ (9)

$$\begin{array}{cc} & =\underset{x_{b}\in {\left\{0,1\right\}}^{m}}{\text{argmin}\;}\;x_{b}^{t}\text{Diag}\left(r^{t}I_{n}⊗p\right)x_{b}+x_{b}^{t}\left(Q⊗P\right)x_{b} \end{array}$$ (10)

$$\begin{array}{cc} & =\underset{x_{b}\in {\left\{0,1\right\}}^{m}}{\text{argmin}\;}\;x_{b}^{t}\left(\text{Diag}\left(r^{t}I_{n}⊗p\right)+Q⊗P\right)x_{b} \end{array}$$ (11)

Here Diag(**v**) refers to the diagonal matrix with entries from **v**. Going from lines (9) to (10) uses the following identity valid for binary vectors: $v^{t}x_{b}=x_{b}^{t}\text{Diag}\left(v\right)x_{b}$.

To summarize, given a number of bits *b*, this procedure approximates the real optimization problem in *n* variables (7) with the QUBO (11) of size *n*⋅*b*. We conclude by remarking that we are not restricted to the cube [−1, 1]^*n*^. If we instead want to optimize over the cube [−*δ*, *δ*]^*n*^, we need to repeat the same construction with the precision vector *δ* ⋅ **p**. An immediate question is how much error is introduced by replacing the cube with a discretized cube.

**Lemma 1**. *Lipschitz Estimate*

*Let* **x**_*T*_ *be an optimal solution to* (7), *and let* **x**_*A*_ *be an optimal solution to* (8). *Then*

$$\begin{array}{c} |r^{t}x_{A}+x_{A}^{t}Qx_{A}-\left(r^{t}x_{T}+x_{T}^{t}Qx_{T}\right)|\leq \sqrt{\frac{n}{2^{b}}}\underset{{\left[-1,1\right]}^{n}}{\text{sup}}\|2Qx+r\| \end{array}$$ (12)

*Proof*. Let $\hat{x_{T}}$ be the point in *C*_*n*,*b*_ closest to **x**_*T*_. The gradient of **r**^*t*^**x** + **x**^*t*^*Q* **x** is 2*Q***x** + **r**, and $\|\hat{x_{T}}-x_{T}\|\leq \sqrt{\frac{n}{2^{b}}}$, leading to the Lipschitz estimate

$$\begin{array}{c} |r^{t}\hat{x_{T}}+{\hat{x_{T}}}^{t}Q\hat{x_{T}}-\left(r^{t}x_{T}+x_{T}^{t}Qx_{T}\right)|\leq \sqrt{\frac{n}{2^{b}}}\underset{{\left[-1,1\right]}^{n}}{\text{sup}}\|2Qx+r\| \end{array}$$ (13)

Combining (13) with the inequality $r^{t}x_{T}+x_{T}^{t}Qx_{T}\leq r^{t}x_{A}+x_{A}^{t}Qx_{A}\leq r^{t}\hat{x_{T}}+{\hat{x_{T}}}^{t}Q\hat{x_{T}}$ we get the following implication:

$$\begin{array}{c} |r^{t}x_{A}+x_{A}^{t}Qx_{A}-\left(r^{t}x_{T}+x_{T}^{t}Qx_{T}\right)|\leq \sqrt{\frac{n}{2^{b}}}\underset{{\left[-1,1\right]}^{n}}{\text{sup}}\|2Qx+r\| \end{array}$$ (14)

Lemma 1 makes a trade-off apparent. With more bits, the solution on the discretized cube will better approximate the true solution of (7) but will require solving a larger QUBO.

Indeed, numerical experiments from subsequent sections will show that *b* = 2 generally requires more iterations than *b* = 8, indicating that the quality of the approximate solution at each step is worse, although interestingly using *b* = 2 takes less time overall since solving smaller QUBOs requires less computational effort. It is also worth noting that estimate (12) does not control the actual distance between solutions, ||**x**_*A*_ − **x**_*T*_||. In the special case when *Q* is positive definite, this distance can be controlled, but it would be interesting to have estimates in greater generality.

The annealers that one works with in practice are never ideal, and so will rarely return the absolute best solution **x**_*A*_ but instead a response consisting of many samples **x**_1_, **x**_2_, …, **x**_*l*_ of good solutions with energies *E*(**x**_*i*_) ≤ *E*(**x**_*i*+1_). (Here *energy* of a solution **x**_*i*_ refers to the value of the objective function at **x**_*i*_). An obvious approach is to treat the lowest energy solution **x**_0_ as the best approximation of **x**_*T*_. A subtler approach that can reap great benefits in practice is to take a linear combination of the full response:

$$\begin{array}{c} \frac{1}{l}\sum_{i=1}^{l}e^{-\beta (E\left(x_{i}\right)-E\left(x_{0}\right))}x_{i} \end{array}$$ (15)

as an approximation of **x**_*T*_, where *β* is a parameter. Experiments in later sections were conducted either using the best response **x**_0_ or the full response with *β* = 100 and performance is compared for several values of *n* and *b*. Although (15) is an *ad hoc* method, the reason for why it works could be due to the energy distribution of the QUBO results. Provided the probability of getting an exited state is Boltzmann distributed (Fermi distribution limit for a single particle at low temperature limits); some responses with considerable low energies could introduce “bit flips” that might correct for the discretization error when added as a linear combination with weights that would decay as an exponential of their energies. A more formal explanation using quantum dynamics for why equation 14 works deserves a further study.

Another approach to get better approximations of **x**_*T*_ is to solve a sequence of QUBOs with bias. More precisely, get an initial approximation of **x**_*T*_ by following the previous procedure to produce $x_{T_{1}}$. Then modify the QUBO by adding a linear term $-\alpha x_{T_{1}}^{t}x$ where *α* > 0 and find approximate solutions to

$$\begin{array}{c} \underset{C_{n,b}}{\text{argmin}\;}\;r^{t}x+x^{t}Qx-\alpha x_{T_{1}}^{t}x=\underset{C_{n,b}}{\text{argmin}\;}\;{\left(r-\alpha x_{T_{1}}\right)}^{t}x+x^{t}Qx \end{array}$$ (16)

By Cauchy-Schwartz, $\frac{x_{T_{1}}}{\|x_{T_{1}}\|}=\underset{\|x\|=1}{\text{argmin}\;}-x_{T_{1}}^{t}x$, thus in solving (16) the annealer is encouraged to produce solutions in the direction of $x_{T_{1}}$. The annealer produces a new lower-energy solution $x_{T_{2}}$ and this process can be repeated until the new solution no longer has lower energy than the previous. Experimental results in later sections contain data with *α* = 0 and.1. Biasing is most helpful in the initial phase of the algorithm when it is iteratively producing solutions close to previous solutions. In later phases biasing is less useful, as will be evident from the results.

### 2.2 An iterative descent algorithm

Algorithms that solve continuous optimization problems rely on a good initial guess and an iterative descent rule. These tasks can be formulated as QUBO problems when trying to minimize the Rayleigh quotient over the unit sphere.

#### 2.2.1 Obtaining an initial guess

Let λ_1_ ≤ λ_2_ ≤ … ≤ λ_*n*_ be the eigenvalues of *A*. To get an initial approximation of λ_1_, one can ask to solve

$$\begin{array}{c} \underset{x\in C_{n,b}}{\text{argmin}\;}\;x^{t}Ax \end{array}$$ (17)

as an approximation of

$$\begin{array}{c} \underset{\|x\|=1}{\text{argmin}\;}\;x^{t}Ax \end{array}$$ (18)

An immediate problem is that if *A* is positive definite, the solution to (17) is just **x** = 0. This can be remedied by replacing *A* with *A* − λ*I*_*n*_, where λ ∈ (λ_*i*_, λ_*i*+1_) for some *i*. The eigenvectors are unaffected, the eigenvalues can be recovered from the new matrix and the solutions to

$$\begin{array}{c} \underset{x\in C_{n,b}}{\text{argmin}\;}\;x^{t}\left(A-\lambda I_{n}\right)x \end{array}$$ (19)

tend to be long, nonzero vectors very close to the span of eigenvectors which have negative eigenvalues for *A* − λ*I*_*n*_, namely **v**_1_, …, **v**_*i*_. A good initial choice is the average of the eigenvalues $\lambda =\frac{tr(A)}{n}$. This is a natural choice since it ensures the initial guess to be within the bounds of the eigenspectrum. As the algorithm progresses, λ will decrease towards λ_1_. To converge in fewer iterations, it’s better to choose λ close to, but greater than λ_1_, as we will examine later.

These observations lead to the following iterative fixed-point method to produce a good initial guess for the lowest eigenvector. Initially solve (19) with $\lambda =\frac{tr(A)}{n}$ to produce a guess **v**_1_. Update λ using the Rayleigh quotient $\lambda =\frac{v_{1}^{t}Av_{1}}{\|v_{1}{\|}^{2}}$ and solve (19) again possibly using **v**_1_ as a bias vector to produce a second guess **v**_2_. Repeat until λ is no longer decreasing.

For small matrices, say 10 × 10, this procedure often suffices to produce the lowest eigenvalue with 2-3 digits of accuracy and the corresponding eigenvector to within a distance of order.1 of the true eigenvector. The descent stage of the algorithm increases the precision to essentially arbitrary order as it will be explained in the next section.

#### 2.2.2 Iterative descent

Suppose we want to minimize a function $f:R^{m}\to R$, and let ∇*f* and *H*(*f*) denote the gradient and Hessian of *f*, respectively. Starting with an initial guess **x**_0_, a common strategy is to Taylor expand *f* around **x**_0_

$$\begin{array}{c} f(x)=f\left(x_{0}\right)+\nabla f\cdot \delta +\delta^{t}\frac{H(f)}{2}\delta +{o(\|\delta \|}^{3}) \end{array}$$ (20)

$$\begin{array}{c} \delta ≔x-x_{0} \end{array}$$ (21)

and choose *δ* to minimize ∇*f* ⋅ *δ*, which is gradient descent, or to minimize $\nabla f\cdot \delta +\delta^{t}\frac{H(f)}{2}\delta$, which includes second order methods such as Newton’s method, BFGS, Newton Conjugate-Gradient, etc. Once a better solution **x**_1_ = **x**_0_ + *δ* has been found, Taylor expand around **x**_1_ again and repeat. The proposed algorithm obtains a good descent direction by using a QUBO to find good approximate solutions to

$$\begin{array}{c} \underset{\delta \in C_{n,b}}{\text{argmin}\;}\;\nabla f\cdot \delta +\delta^{t}\frac{H(f)}{2}\delta \end{array}$$

Similar to Newton-CG and BFGS, this method requires computing, but not inverting, the Hessian matrix, and benefits from a line search which possibly increases the size of *δ*. See [10] for more details on classical optimization algorithms and the benefits of line search. Here the line search step amounts to minimizing a quadratic expression, and so the optimal scaling can be directly computed.

If *k*^*th*^ approximate solution **x**_*k*_ is closer to the true solution than any point in the discretized cube, one cannot expect minimizing the QUBO to produce a better solution. A key part of the descent phase is enforcing a minimum step size in addition to the line search so that the candidate *k* + 1^*st*^ solution is possibly *worse* than that *k*^*th*^. If the candidate solution is worse, the algorithm discards the candidate and replaces the discretized unit cube *C*_*n*,*b*_ by a scaled-down discretized cube *t* ⋅ *C*_*n*,*b*_ where *t* ≪ 1, which amounts to repeating the procedure outlined in section 3 with the precision vector *t* ⋅ **p**. Once the discretized cube has been scaled down, the algorithm continues running until it needs to scale down the cube further, or exits having achieved the desired accuracy.

### 2.3 The algorithm

With the key ingredients covered, we are in a position to present the algorithm. As a reminder, at each step, the objective function is of the form *f*(**x**) = **x**^*t*^(*A* − λ*I*_*n*_)**x**, whose gradient and Hessian can be calculated as ∇*f* = 2(*A* − λ*I*_*n*_)**x**^*t*^ and *H*(*f*) = 2(*A* − λ*I*_*n*_) respectively. These formulas are implicitly used in the descent phase of the algorithm. At several stages, the algorithm solves optimization problems of the form $\underset{x\in t\cdot C_{n,b}}{\text{argmin}}\;r^{t}x+x^{t}Ax$. These are turned in to QUBOs as explained in section 2.1, and annealers are used to minimize the QUBOs that appear, possibly using full responses or biasing. In subsequent section the effects of biasing, full responses and other parameters will be analyzed.

**Algorithm 1** Controlled Precision QUBO-based Algorithm to Compute Eigenvectors of Symmetric Matrices

 Inputs: Symmetric *n* × *n* matrix *A*, bits for precision vector *b*, desired precision *ϵ*_*tol*_

 Outputs: Smallest evec **v** and eval λ within *ϵ*_*tol*_ of true values

 $\lambda \leftarrow \frac{tr(A)}{n}$

 *H* ← *A* − λ*I*_*n*_       // Enforcing Indefiniteness

 $v\leftarrow \underset{C_{n,b}}{\text{argmin}}\;x^{t}Hx$         // Initial Guess Phase

 $v\leftarrow \frac{v}{\left\|v\right\|}$

 **while v**^*t*^*A***v** < λ **do**

  $\lambda \leftarrow \frac{v^{t}Av}{{\left\|v\right\|}^{2}}$

  *H* ← *A* − λ ⋅ *I*_*n*_

  $v\leftarrow \underset{C_{n,b}}{\text{argmin}\;}x^{t}Hx$

  $v\leftarrow \frac{v}{\left\|v\right\|}$

 **end while**

 *precision* ← .1    // Descent Phase

 **while** *precision* > *ϵ*_*tol*_ **do**

  *H* ← *A* − λ*I*_*n*_

  $\delta \leftarrow \underset{precision\cdot C_{n,b}}{\text{argmin}\;}2v^{t}H\delta +\delta H\delta$       // Getting Descent Direction

  *δ* ← *δ* − 〈**v**, *δ*〉*δ*    // Orthogonalizing *δ*, **v**

  *t*_*min*_ = max{−**v**^*t*^*Hδ*/(*δ*^*t*^*Hδ*), 1}    // Line search step

  *δ* ← *t*_*min*_ ⋅ *δ*

  **if** $\frac{{\left(v+\delta \right)}^{t}A\left(v+\delta \right)}{\|v+{\delta \|}^{2}}<\lambda$ **then**    // Checking if solution improves

   $v\leftarrow \frac{v+\delta }{\left\|v+\delta \right\|}$

   λ ← **v**^*t*^*A***v**

  **else**

   *precision* ← .1 ⋅ *precision*    // Increasing precision otherwise

  **end if**

 **end while**

 **return v**, λ

Two steps merit a bit more explanation. Replacing *δ* by *δ* − 〈**v**, *δ*〉*δ* forces **v**, *δ* to be orthogonal. Since the Rayleigh quotient needs to be optimized over the sphere, the update direction *δ* should be tangent to the sphere at **v**, and the tangent space of the sphere at **v** is precisely the set of vectors orthogonal to **v**. Second, the scaling *δ* is computed as *t*_*min*_ = max{−**v**^*t*^*Hδ*/(*δ*^*t*^*Hδ*), 1}. The expression −**v**^*t*^*Hδ*/(*δ*^*t*^*Hδ*) is the line search step coming from minimizing the quadratic (**v** + *t*_*min*_*δ*)^*t*^*H*(**v** + *t*_*min*_*δ*). Strictly speaking this quadratic expression only has a minimum when *δ*^*t*^*Hδ* is positive, and in practice when using this algorithm it almost always is, and if not, set *t*_*min*_ = 1. A minimum scaling *t*_*min*_ ≥ 1 is enforced so that the candidate update **v** + *t*_*min*_*δ* possibly overshoots the exact solution, resulting in a worse estimate of the lowest eigenvector. Overshooting is an indication that the discretized cube is no longer fine enough to produce better solutions, and so the candidate update is discarded and the discretized cube is scaled down. Intuitively the scaling at each step should be about the order of $\frac{1}{2^{b-1}}$, and the numerical experiments below all use a.1 factor.

Oftentimes in practice one wishes to solve a generalized eigenvalue problem of the form *A***v** = λ*B***v**. In the case when *A*, *B* are symmetric and *B* is strictly positive definite, the smallest generalized eigenvalue minimizes the generalized Rayleigh quotient

$$\begin{array}{c} \lambda =\underset{\|x\|=1}{\text{min}}\;\frac{x^{t}Ax}{x^{t}Bx}\;\;v=\underset{\|x\|=1}{\text{argmin}\;}\;\frac{x^{t}Ax}{x^{t}Bx} \end{array}$$ (22)

The following small changes solves the generalized eigenvalue problem, again to essentially arbitrary precision. First, instead of initializing λ as $\frac{tr(A)}{n}$, one can generate a random unit vector **w** (or use a specified vector) and initialize $\lambda =\frac{w^{t}Aw}{w^{t}Bw}$. Second, replace every Rayleigh quotient with the corresponding generalized Rayleigh quotient. Lastly, instead of updating *H* as *H* = *A* − λ*I*_*n*_, update as *H* = *A* − λ*B*, as the latter preserves the *B*-eigenspectrum of *A*, while the former does not.

We conclude by emphasizing that this algorithm reaches arbitrary precision without increasing the size of the QUBOs, all of which involve *n* ⋅ *b* binary variables. Additionally, all quadratic problems are of the form **r**^*t*^ **x** + **x**^*t*^(*A* − λ*I*_*n*_)**x** where *A* is fixed, implying that the potential non-zero coefficients of the QUBO do not change (examine formula (11)).

## 3 Experimental results

The algorithm and its variants are tested on a class of random matrices of varying sizes and on benchmark sparse matrices using D-Wave’s simulated annealing (SA) software unless otherwise specified. For each experiment, the algorithm ran until the Rayleigh quotient of the approximate eigenvector was within 10^−8^ of the true value. The *total annealing time* is hence computed as the total amount of time the algorithm spends on performing SA. The SA is the bottle-neck for the algorithm, and we include the time to give the reader a sense of how the run-time varies with the size of the matrix *n* and the number of bits *b*, as we will demonstrate in subsequent sections. Adjusting the time for each anneal will not affect the accuracy of the algorithm, as it can reach desired precision with any reasonably good annealer, but may affect the run-time. For a specific architecture, one might be able to tune the anneal time optimally based on *n* and *b*, which is not a question we attempt to answer here.

### 3.1 Basic performance

**Fig 1:**
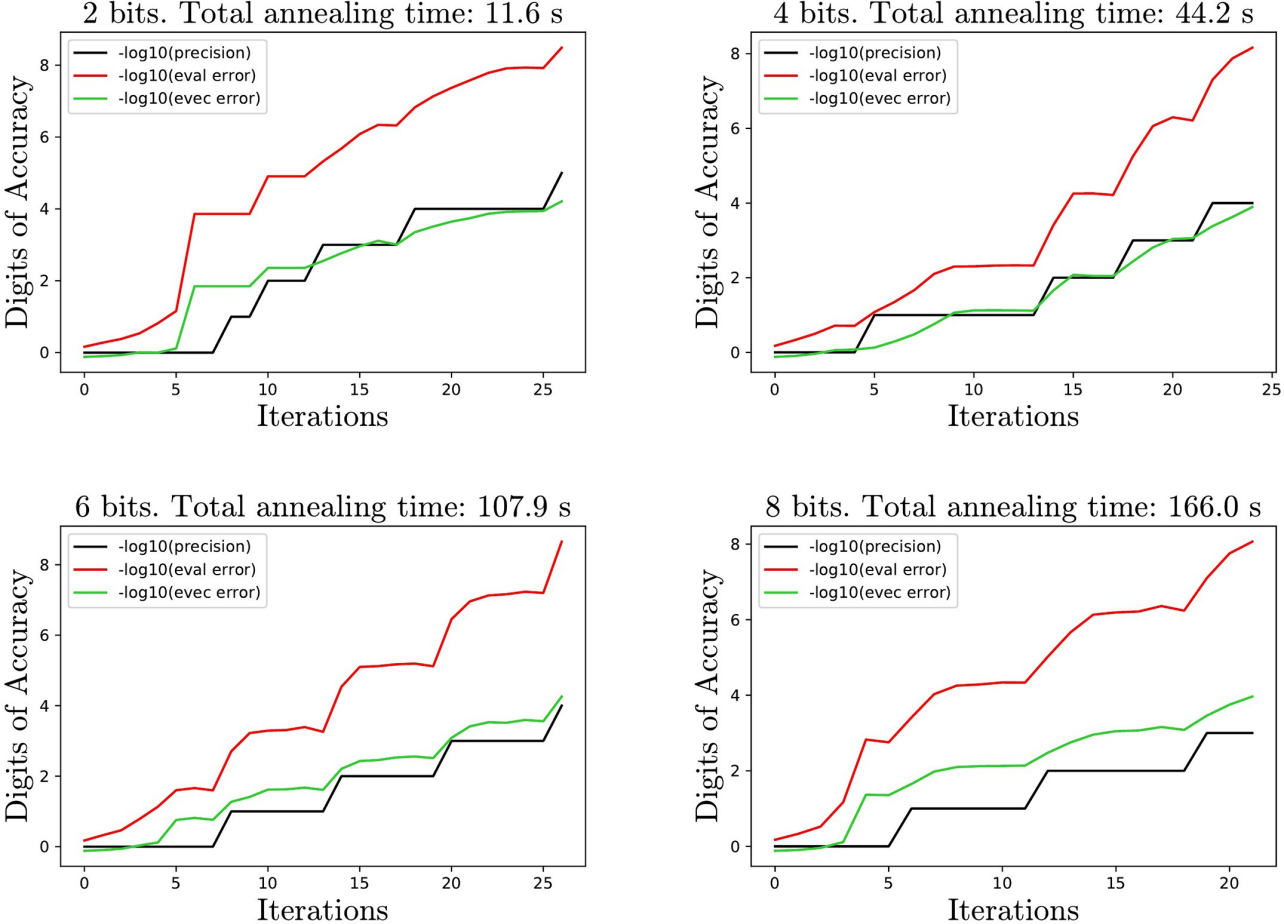
Digits of accuracy plotted against number of iterations. *Total annealing time* is the total amount of time the algorithm spends on performing SA, since SA dominates the cost of the method. *Evec error* is the distance of the computed vector from the true unit eigenvector. *Precision* refers to the scaling applied to the discretized cube at each iteration. The initial guess phase corresponds to a precision of 1. Observe that whenever the error increases, the algorithm responds by increasing the precision, which often gives large accuracy gains within the subsequent 2-3 iterations.

First, we demonstrate the convergence as a function of the number of iterations using example matrices from the TAMU SuiteSparse collection [14]. Fig 1 shows performance on the breasttissue_10NN matrix, a weighted graph adjacency matrix of size 106 × 106 for 2, 4, 6 and 8 bits using best response and no biasing.

**Fig 2:**
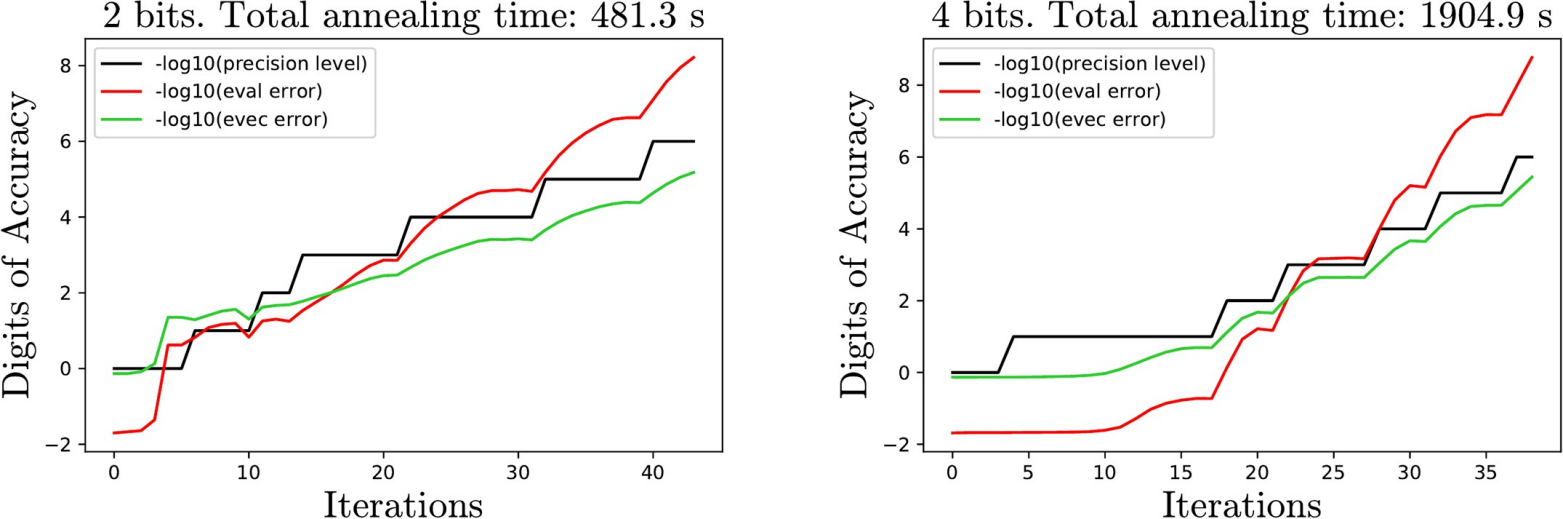
Error plot for 560 × 560 space Shuttle control matrix.

Interestingly using fewer bits gives less time to reach desired accuracy despite requiring more iterations. A *log* − *log* regression on the MP matrices (see next section) gives that the anneal time grows like (*n* ⋅ *b*)^1.57^ and the number of iterations grows like *n*^.44^*b*^−.32^ so the total time is roughly *n*^2^*b*^1.2^. The algorithm works on even larger matrices, as is demonstrated in Fig 2 using the spaceShuttleEntry_1 matrix, a 560 × 560 control matrix.

**Fig 3:**
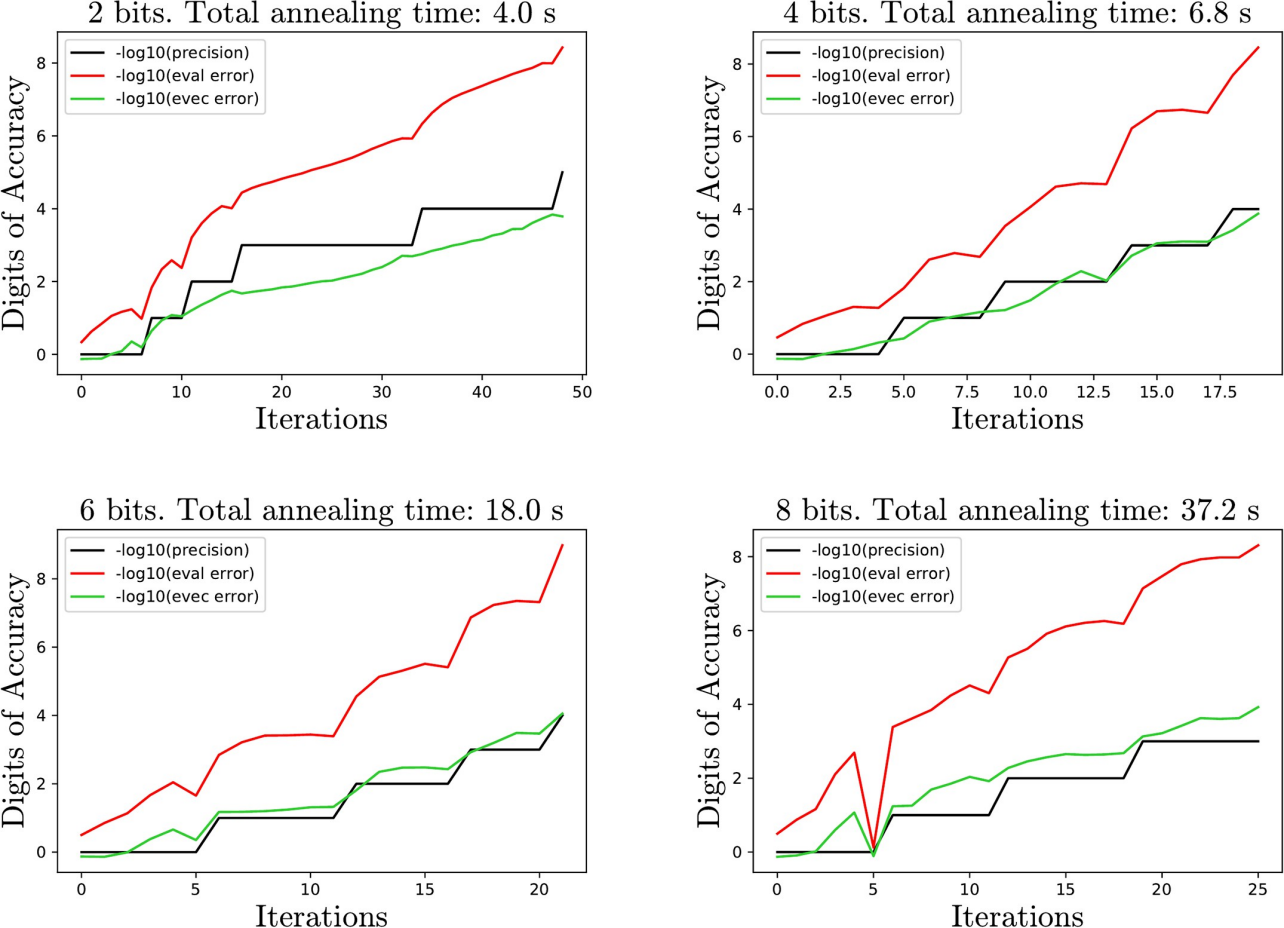
Error plots for generalized eigenvalue problem on two 48 × 48 mesh matrices.

Fig 3 demonstrates the performance for the generalized eigenvalue problems using mesh1em1 as the *A* matrix and meshe1 as the *B* matrix, two 48 × 48 matrices from the SuiteSparse database.

In order to try to get algorithms that run as fast as possible, one might ask if it is possible get the algorithm to work using 1 bit of precision. With the current scheme this cannot be done. However, by reformulating the problem as an Ising instead of QUBO, one can indeed use only one bit precision. Ising problems are of the form

$$\begin{array}{c} \underset{x\in {\left\{-1,1\right\}}^{n}}{\text{argmin}}\;h^{t}x+x^{t}Qx \end{array}$$

the main distinction from QUBOs being the spin variables ±1. QUBOs or Ising problems are mathematically equivalent, and most annealers are capable of solving either.

Using the Ising formulation, its possible to mimic the same algorithm, which works well on very small matrices. However, for larger matrices, such as for the 106 × 106 weighted adjacency matrix the single-bit version of the algorithm takes longer than using two bits, taking 32.3 seconds and requiring over 400 iterations (compare with Fig 1). An educated guess for why this might happen follows. Since the solutions produced by Ising problems have coordinates that are all non-zero and of the same magnitude, if the algorithm has already produced a solution whose *k*^*th*^ coordinate is close to the true value, the added solution from the Ising problem will force that coordinate away from the optimal value. Using two bits is effective because the solutions can have coordinates that are positive, negative, or zero.

### 3.2 Analysis of the parameters

**Fig 4:**
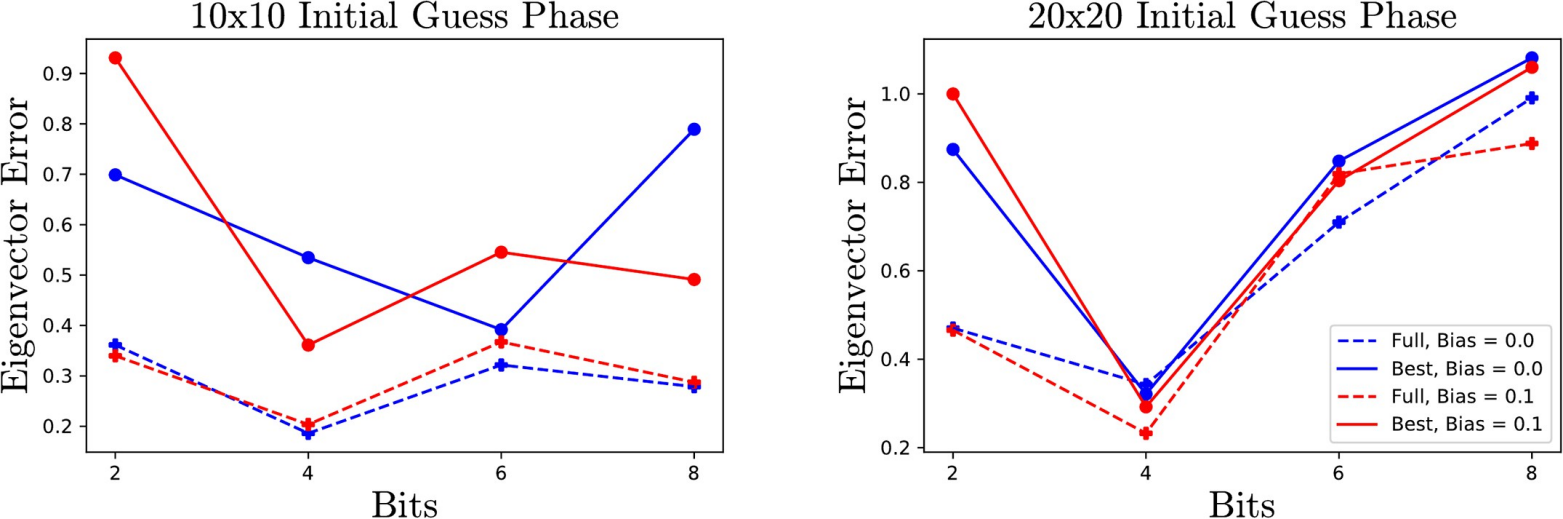
Eigenvector error for MP matrices at the end of initial guess phase. Observe that for small QUBOs the full response decreases the error significantly.

**Fig 5:**
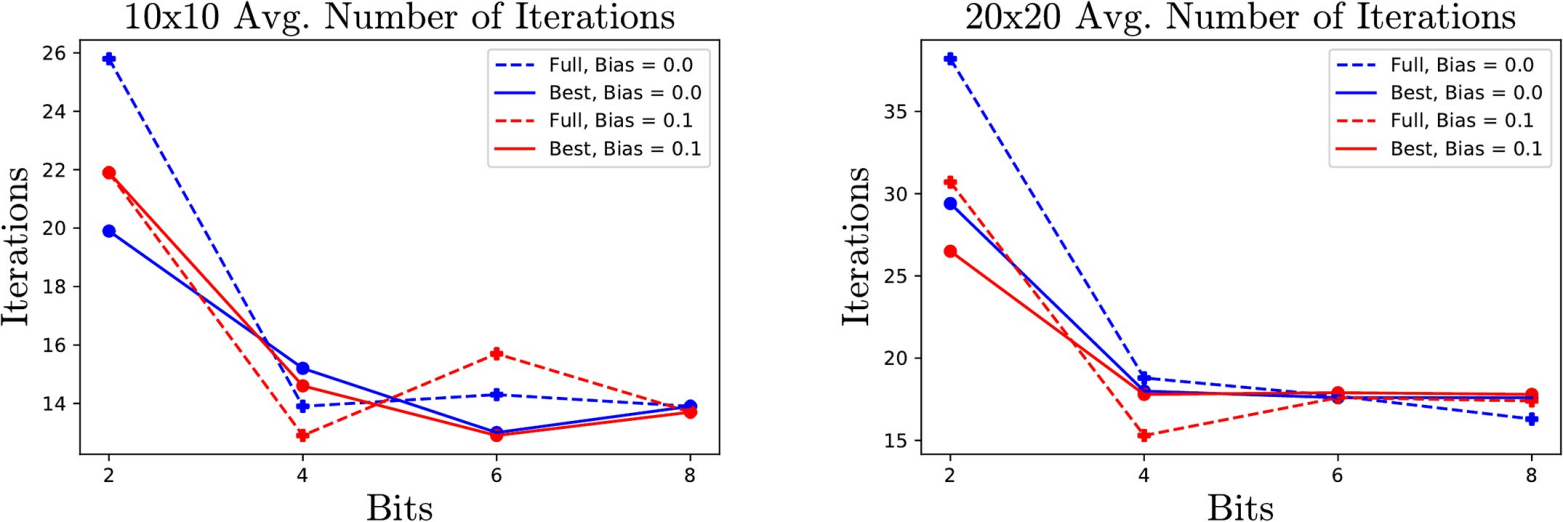
Average number of iterations required for MP matrices for different parameters.

To demonstrate the effect of biasing and full response parameters, the algorithm is tested on small matrices of sizes 3, 10 and 20 with number of bits *b* = 2, 4, 6 and 8. In the interest of not overwhelming the reader with plots and data, only the data for matrices of size 10 and 20 is displayed. We analyze the error at the end of the initial guess phase, and the average number of iterations each method requires. For each choice of size, bits and parameters, 10 Marchenko-Pasture matrices [15] with parameter λ = .3 are generated and the average errors at the end of the descent phase is recorded. Ideally this initial phase should end with the smallest possible error before beginning the descent phase. Towards this end, taking full responses (Eq (15)) and biases (Eq (16)) can be very beneficial. However, this benefit fades as the sizes of the QUBOs increase as one can see from Figs 4 and 5.

**Fig 6:**
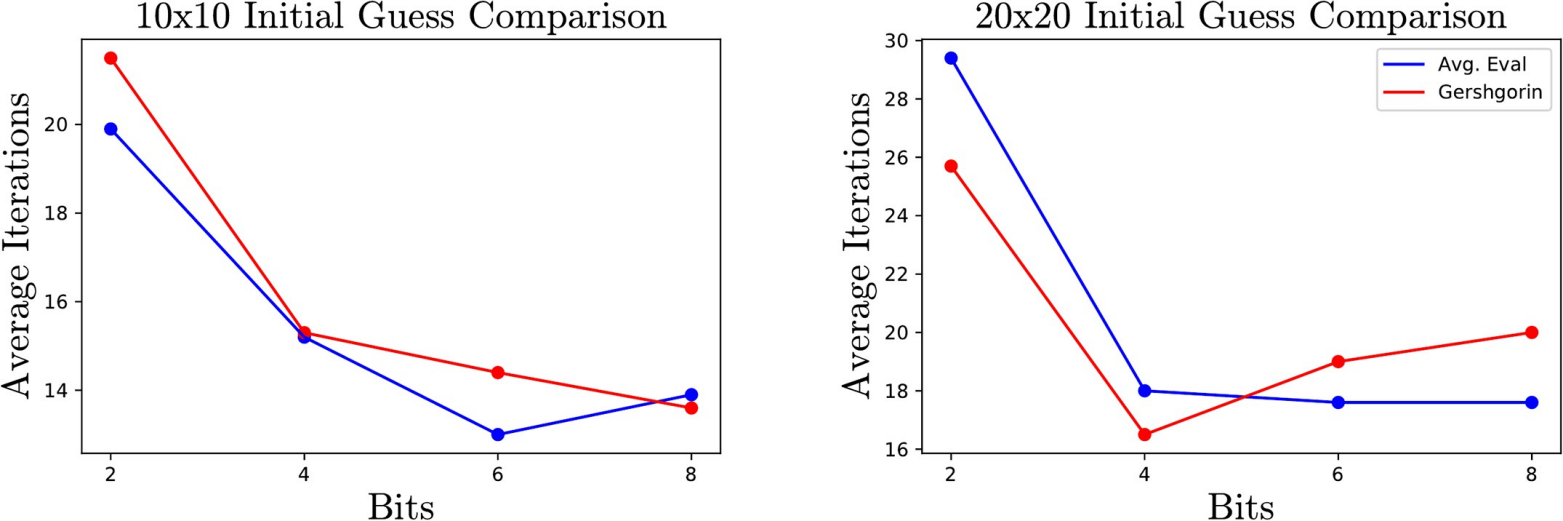
Total iterations required for different initializations of λ.

The choice to initialize λ as $\frac{tr(A)}{n}$ is motivated by a desire to produce an initial guess which is close to, but greater than, the true lowest eigenvector. To demonstrate this effect on 10 × 10 and 20 × 20 matrices, we compare performance initializing as $\frac{tr(A)}{n}$, which is the average of all the eigenvalues against initializing as the highest Gershgorin bound, which upper bounds the maximum eigenvalue [16]. Choosing an initialization closer to the true eigenvalue often leads to fewer iterations, although the difference is somewhat small and fades as the number of bits increases, as seen in Fig 6.

### 3.3 Gap size analysis

**Fig 7:**
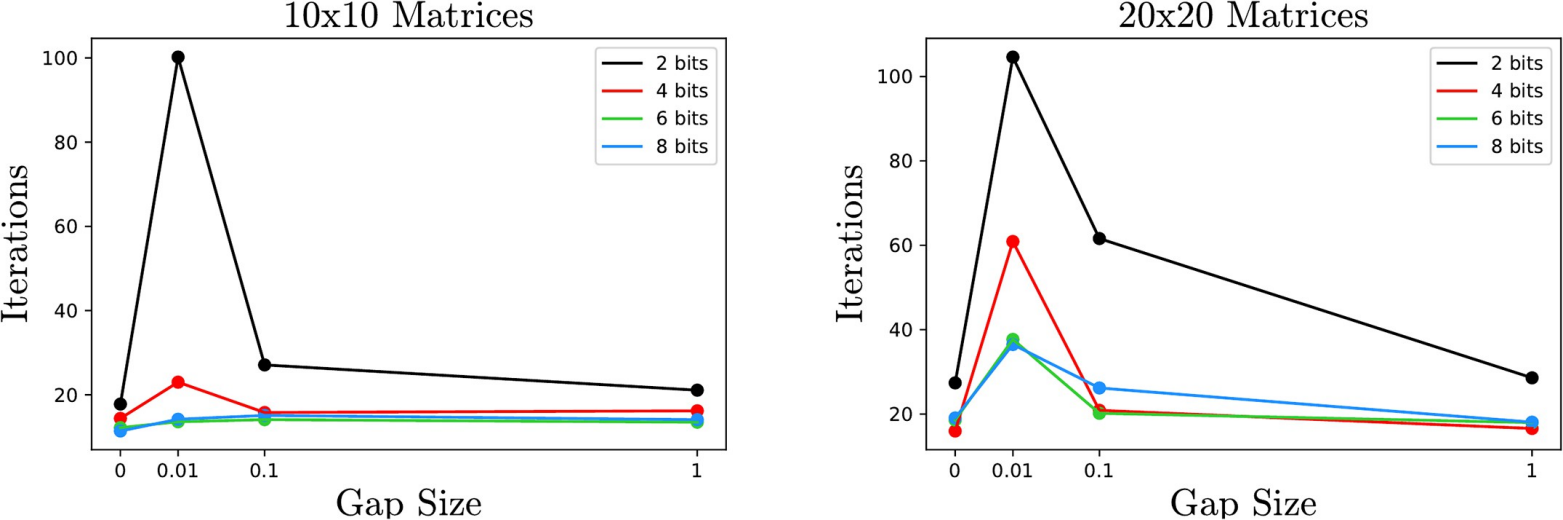
Average number of iterations on 30 samples as a function of the gap size |λ_1_ − λ_2_|. The smaller the gap, the more iterations required, especially when the number of bits is small. In the extreme case where the gap is 0 and the lowest eigenvalue appears with multiplicity, the algorithm is actually faster in the sense that fewer iterations are needed for the computed eigenvalue to approximate the true eigenvalue.

Here we analyze the effect of the spacing between eigenvalues. In particular, the gap |λ_1_ − λ_2_| can significantly affect the number of iterations required to reach a given precision. For this experiment, given a gap size *g* = |λ_1_ − λ_2_|, an orthogonal matrix *U* is chosen at random with respect to the Haar measure using the SciPy implementation of [17]. The algorithm is then analyzed on the matrix *U*^*t*^Diag(0, *g*, 1, …, *n* − 2)*U*. As Fig 7 demonstrates, as the gap size decreases the algorithm takes longer to achieve a given accuracy. The exception is when the gap is 0, and the smallest eigenvalue appears with multiplicity. In this case the algorithm actually requires fewer iterations.

**Fig 8:**
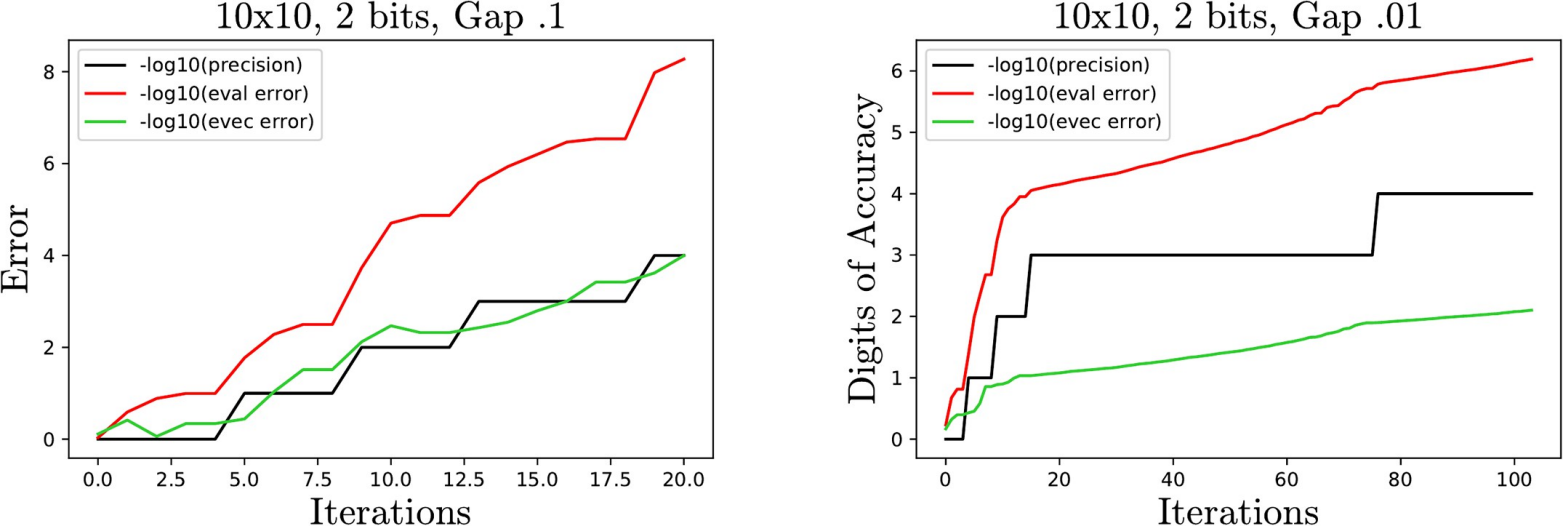
Sample plots for two 10 × 10 matrices with gap size .1 and .01. As the gap size decreases, the ratio of eigenvector error to precision and eigenvalue error increases.

Fig 8 has two example error plots demonstrating the slower convergence. Observe that the eigenvector error relative to both the precision and eigenvalue error increases as the gap size decreases.

**Fig 9:**
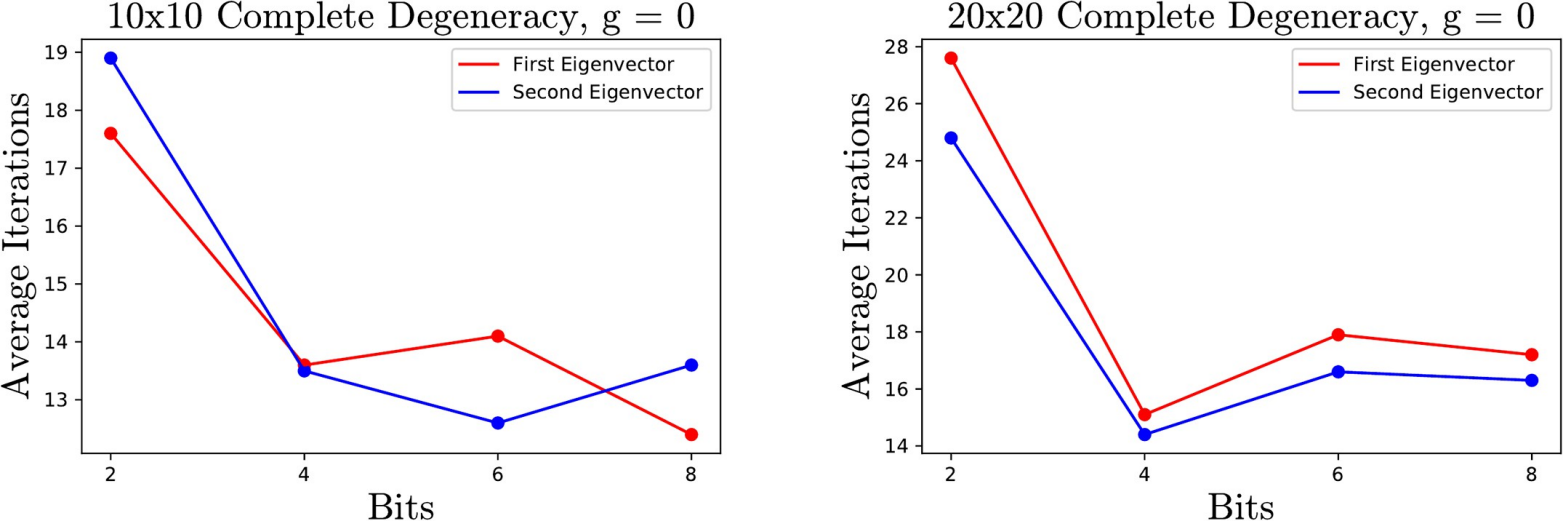
Average number of iterations running the algorithm twice on matrices with complete degeneracy. One can see slightly better performance on the second run, particularly on the 20 × 20 matrices.

In the case when there is degeneracy, that is the gap *g* is 0, one might want two eigenvectors that span the eigenspace. This can be accomplished by running the algorithm once to get an approximate eigenvector **v**_1_, replace the matrix *A* with $A+\alpha v_{1}v_{1}^{t}$ where *α* > 0, and run the algorithm again to get the eigenvector **v**_2_. By the spectral theorem for the symmetric matrix $A+\alpha v_{1}v_{1}^{t}$, $v_{1}^{t}v_{2}=0$ implying that **v**_2_ is an eigenvector for *A*. Replacing *A* by $A+\alpha v_{1}v_{1}^{t}$ is necessary for numeric purposes. The gap *g* is never numerically zero, so if the algorithm is run twice on the matrix *A* even with different randomization, it will often produce the same vector. One can also try to take advantage of the first computation by initializing the approximate eigenvalue to λ_*n*_ + *ϵ* in the second run of the algorithm. The data shown below in Fig 9 was collected for $\alpha =\frac{tr(A)}{n}-\lambda_{n}$ and *ϵ* = 1, and one can see a slight boost in performance in the second run of the algorithm.

## 4 Conclusion

We have proposed and tested an algorithm to find eigenvectors of symmetric matrices by minimizing the corresponding Rayleigh quotient with an iterative steepest-descent method. Initial guesses and subsequent descent directions are found by looking for minima over discretized cubes of various sizes, encoded as QUBO problem which is in turn solved with a SA method. The algorithm is able to reach essentially arbitrary precision even for fairly large matrices. We have performed a thorough study of the effect of the different parameters, including, the eigenvalue spacing, initial guesses, number of bits, and the matrix size. We have explored the possibility of using a single bit precision by reformulating the QUBO problem as an Ising problem. Finally, we have introduced two novel approaches to accelerate the convergence such as biasing and using a larger set of solution from the SA step. These two approaches might be applicable to other QUBO based problems. We encourage the reader to test these algorithms on other annealing devices.
